# Synergistic Activity of Fosfomycin, Ciprofloxacin, and Gentamicin Against *Escherichia coli* and *Pseudomonas aeruginosa* Biofilms

**DOI:** 10.3389/fmicb.2019.02522

**Published:** 2019-11-06

**Authors:** Lei Wang, Mariagrazia Di Luca, Tamta Tkhilaishvili, Andrej Trampuz, Mercedes Gonzalez Moreno

**Affiliations:** ^1^Center for Musculoskeletal Surgery, Charité – Universitätsmedizin Berlin, Corporate Member of Freie Universität Berlin, Humboldt-Universität zu Berlin and Berlin Institute of Health, Berlin, Germany; ^2^Berlin-Brandenburg Center for Regenerative Therapies, Charité – Universitätsmedizin Berlin, Berlin, Germany

**Keywords:** *Escherichia coli*, *Pseudomonas aeruginosa*, biofilm-associated infection, antibiotic activity, synergism, clinical isolates, antibiotic resistance, isothermal microcalorimetry

## Abstract

Gram-negative (GN) rods cause about 10% periprosthetic joint infection (PJI) and represent an increasing challenge due to emergence of antimicrobial resistance. *Escherichia coli* and *Pseudomonas aeruginosa* are among the most common cause of GN-PJI and ciprofloxacin is the first-line antibiotic. Due to emergence of fluoroquinolone resistance, we evaluated *in vitro* the activity of fosfomycin, ciprofloxacin, and gentamicin, alone and in combinations, against *E. coli* and *P. aeruginosa* biofilms. Conventional microbiological tests and isothermal microcalorimetry were applied to investigate the anti-biofilm activity of the selected antibiotics against standard laboratory strains as well as clinical strains isolated from patients with prosthetic joint associated infections. The biofilm susceptibility to each antibiotic varied widely among strains, while fosfomycin presented a poor anti-biofilm activity against *P. aeruginosa*. Synergism of two-pair antibiotic combinations was observed against different clinical strains from both species. Highest synergism was found for the fosfomycin/gentamicin combination against the biofilm of *E. coli* strains (75%), including a gentamicin-resistant but fosfomycin-susceptible strain, whereas the gentamicin/ciprofloxacin combination presented synergism with higher frequency against the biofilm of *P. aeruginosa* strains (71.4%). A hypothetical bacteriolysis effect of gentamicin could explain why combinations with this antibiotic seem to be particularly effective. Still, the underlying mechanism of the synergistic effect on biofilms is unknown. In conclusion, combinatorial antibiotic application has shown to be more effective against biofilms compared to monotherapy. Further *in vivo* and clinical studies are essential to define the potential treatment regimen based on our results.

## Introduction

Gram-negative (GN) rods cause about 10% of periprosthetic joint infection (PJI) and represent an increasing treatment challenge due to the emergence of resistance worldwide (Shah et al., 2016; Thompson et al., 2018). Enterobacteriaceae are most frequently isolated in GN-PJI, followed by *Pseudomonas aeruginosa* (Fantoni et al., 2019). GN-PJI can occur after hematogenous seeding from a distant infectious focus (i.e. urinary or intestinal tract) or can be introduced during arthroplasty and manifest in the early postoperative period (Sendi et al., 2010). Over a period of a few years (2003–2012), the occurrence of PJIs due to multidrug-resistant GN bacteria has increased significantly, in the case of *E. coli*, from 2 to 4.3%, and for *P. aeruginosa*, from 0.7 to 1.8% (Benito et al., 2016). Antimicrobial resistance in GN rods is increasing at both, community and hospital levels, and is often associated with treatment failure (Virginio et al., 2019). The worldwide rise of carbapenem-resistant GN bacilli is of major concern for the public health. While formally this problem was mostly related to *Pseudomonas* and *Acinetobacter* species, the rising trend in *E. coli* may lead to almost untreatable community-acquired infections (Tangden and Giske, 2015).

In spite of its vast impact on patients and the health-care system (Haddad et al., 2017), the management of GN-PJI is difficult due to the lack of a “gold standard” treatment strategy (Zmistowski et al., 2011; Goel et al., 2017). In fluoroquinolone-susceptible GN rods, ciprofloxacin is recommended for PJI (Sousa and Abreu, 2018). However, the growing quinolone-resistance in GN bacteria makes the treatment of GN-PJI more challenging complicating the clinical outcome (Rodriguez-Pardo et al., 2014; Benito et al., 2016). Due to unmet medical needs of currently available antibiotics, combination therapy has been investigated as an alternative strategy for GN-PJI treatment (Taha et al., 2018). Especially, revival of older antibiotics such as fosfomycin gained attention for treatment of multi-drug resistant GN rods (Walsh et al., 2016).

Beside antimicrobial resistance, treatment of PJI is challenged by the microbial persistence on the surface of implants forming biofilms (Taha et al., 2018). In biofilms, microbes exhibit “phenotypical resistance” to standard antibiotics (Donlan and Costerton, 2002). Therefore, it is essential to look at possible anti-biofilm activities of single or combined antibiotics. Several pre-clinical investigations of fosfomycin combination therapy have shown synergistic activity against biofilms of GN bacteria (Falagas et al., 2016), particularly in combination with fluoroquinolones or aminoglycosides (Michalopoulos et al., 2011; Corvec et al., 2013). Nevertheless, no systematic studies investigated these combinations against *P. aeruginosa* and *Escherichia coli* biofilms in the same experimental settings. Thus, we focused on combinations involving fosfomycin, ciprofloxacin, and gentamicin as representatives of the above-mentioned antibiotic classes. These three antibiotics present a bactericidal effect against bacteria showing different mechanisms of action. Fosfomycin has a unique mode of action inhibiting irreversibly an early stage of bacterial cell wall biosynthesis (Dijkmans et al., 2017), whereas ciprofloxacin inhibits bacterial DNA replication (Thai and Zito, 2019) and gentamicin inhibits the bacterial protein synthesis (Kumar et al., 2008).

Accurate experimental data from the investigation of combinatorial therapy with paired antibiotics might bring new evidences on their potential in the treatment of GN-PJI. Hence, we evaluated the *in vitro* activity of single and combinations of fosfomycin, ciprofloxacin, and gentamicin against planktonic and biofilms of *P. aeruginosa* and *E. coli* strains, including resistant clinical isolates obtained from patients with prosthetic joint associated infections, by using conventional microbiological tests and isothermal microcalorimetry.

## Materials and Methods

### Bacterial Strains

*E. coli* (ATCC 25922) and *P. aeruginosa* (ATCC 27853) laboratory standard strains were used in this study. Moreover, eight *E. coli* and seven *P. aeruginosa* clinical isolates obtained from consecutive patients diagnosed with PJI between 2015 and 2017 were used for this study. For the diagnosis of PJI, the PRO-IMPLANT diagnostic criteria were used (Li et al., 2018; Izakovicova et al., 2019). The clinical isolates were used from the biobank collection, which is part of the prospective institutional PJI cohort. The study was approved by the institutional ethical committee (EA1/040/14) and was conducted in accordance with the most recent iteration of the Declaration of Helsinki. According to the ethical approval, participants’ informed consent was waived and all data were pseudonymized. Bacteria were stored at −80°C using a cryovial bead preservation system (Microbank; Pro-Lab Diagnostics, Canada).

### Antimicrobial Agents

Fosfomycin (5 g; InfectoPharm, Heppenheim, Germany) was provided as purified powder by the manufacturer. Ciprofloxacin injectable solution (2 mg/ml; Fresenius Kabi GmbH, Bad Homburg, Germany) and gentamicin injectable solution (40 mg/ml; Ratiopharm GmbH, Ulm, Germany) were purchased from the respective manufacturers. Stock solutions of appropriate concentrations were prepared in sterile 0.9% saline.

### Etest

Etest (bioMerieux, Marcy-l’Étoile, France) was performed in Mueller-Hinton agar (MHA) (Becton, Dickinson and Company, Germany) following the manufacturer’s instructions. The minimum inhibitory concentration (MIC) was determined as the concentration at which the inhibition ellipse intersected the scale of the strip after incubation at 37°C for 24 h. To evaluate the susceptibility, the antimicrobial susceptibility breakpoints from the CLSI (CLSI, 2015) were used. All experiments were performed in triplicates.

### Broth Macrodilution Assays

The MIC and the minimum bactericidal concentration (MBC) phase were determined for fosfomycin, ciprofloxacin, and gentamicin by the broth macrodilution assay (BMD) in cation-adjusted Mueller-Hinton broth (CAMHB) (BD, Le Pont de Claix, France), according to the Clinical and Laboratory Standards Institute (CLSI) guidelines (CLSI, 1999). An inoculum of approximately 5 × 10^5^ CFU/ml were used. Two-fold serial dilutions of each antibiotic were prepared in 1 ml medium in plastic tubes and incubated for 24 h at 37°C. The MIC was defined as the lowest concentration of antibiotic that completely inhibited visible growth.

After the incubation, all tubes without visible growth were vigorously vortexed, aliquots of 100 μl were plated on Tryptic Soy Agar (TSA) (Oxoid, Basingstoke, UK) plates, and the numbers of bacteria were determined. The MBC was defined as the lowest antimicrobial concentration that killed ≥99.9% of the initial bacterial inoculum after 24 h. The medium was supplemented with 25 mg/L glucose-6-phosphate for testing of fosfomycin. Glucose-6-phosphate induces the transport system *via* which fosfomycin is actively absorbed into the bacteria (Dijkmans et al., 2017). All experiments were performed in triplicates.

### Assessment of Antimicrobial Activity by Isothermal Microcalorimetry and Sonication/Colony-Counting

The antimicrobial activity of fosfomycin, ciprofloxacin, and gentamicin against either *E. coli* or *P. aeruginosa* ATCC strains was determined by isothermal microcalorimetry (IMC) as described previously (Butini et al., 2018). Briefly, planktonic bacteria (5 × 10^5^ CFU/ml) were treated with serial dilutions of antibiotics in CAMHB, and production of heat was measured for 24 h. The minimum heat inhibitory concentration (MHIC) was defined as the lowest concentration of antibiotic able to suppress the metabolic heat production of planktonic bacteria.

*E. coli* and *P. aeruginosa* biofilm formation was assessed by incubating porous glass beads (ROBU®, Hattert, Germany) in inoculated CAMHB with 2–3 colonies of the corresponding bacteria at 37°C. The ratio between beads and diluted bacterial suspension was 1 bead:1 ml, with a maximum of 10 beads per 50 ml Falcon tube. After 24 h incubation, beads were washed three times with sterile 0.9% saline to remove planktonic bacteria and exposed to serial dilutions of antibiotic in 1 ml of CAMHB and incubated for a further 24 h at 37°C. The media were supplemented with 25 mg/L glucose-6-phosphate for fosfomycin testing. After exposure to antibiotics, beads were washed three times with 0.9% saline, placed in glass ampoules containing 3 ml of CAMHB and introduced into the calorimeter (thermal activity monitor, model 3102 TAM III; TA Instruments, New Castle, USA). Sterile beads were used as negative control. Production of heat was recorded for 48 h to detect bacterial activity. The minimum biofilm bactericidal concentration (MBBC) was defined as the lowest concentration of antibiotic that strongly reduced biofilm cells viability and led to the absence of heat flow production after 48 h of incubation at 37°C.

Moreover, the biofilm-eradicating activity of these three antibiotics on clinical isolates from both species was evaluated by sonication and colony-counting as in a previous study (Gonzalez Moreno et al., 2019). The minimum biofilm eradicating concentration (MBEC) was defined as the lowest concentration of antibiotic required to kill all sessile cells resulting in the appearance of no colony after plating sonication fluid (detection limit: <20 CFU/ml).

The synergistic effect of antibiotic combinations was evaluated against both ATCC species following the IMC assay as described above and through CFU counting of the sonicated beads. The synergistic activity was evaluated by calculation of the fractional biofilm eradication concentration index (FBECI) as described in a previous study (Dall et al., 2018), where a FBECI of ≤0.5 indicates a synergistic effect. The FBECI was calculated following the equation: FBECI = FBECI A + FBECI B = MBEC_A_ combination/MBEC_A_ alone + MBEC_B_ combination/MBEC_B_ alone, where MBEC_A_ combination and MBEC_B_ combination are the MBEC of compound A in the presence of B and compound B in the presence of A, respectively; MBEC_A_ alone and MBEC_B_ alone are the FBECI of compound A and compound B, respectively.

Data from IMC were analyzed by the manufacturer’s software (TAM Assistant; TA Instruments) and Prism 7.0 (GraphPad Software, La Jolla, CA). All experiments were performed in triplicates.

## Results

### Activity of Antibiotics Against *E. coli* and *P. aeruginosa* ATCC Strains

**Figure 1:**
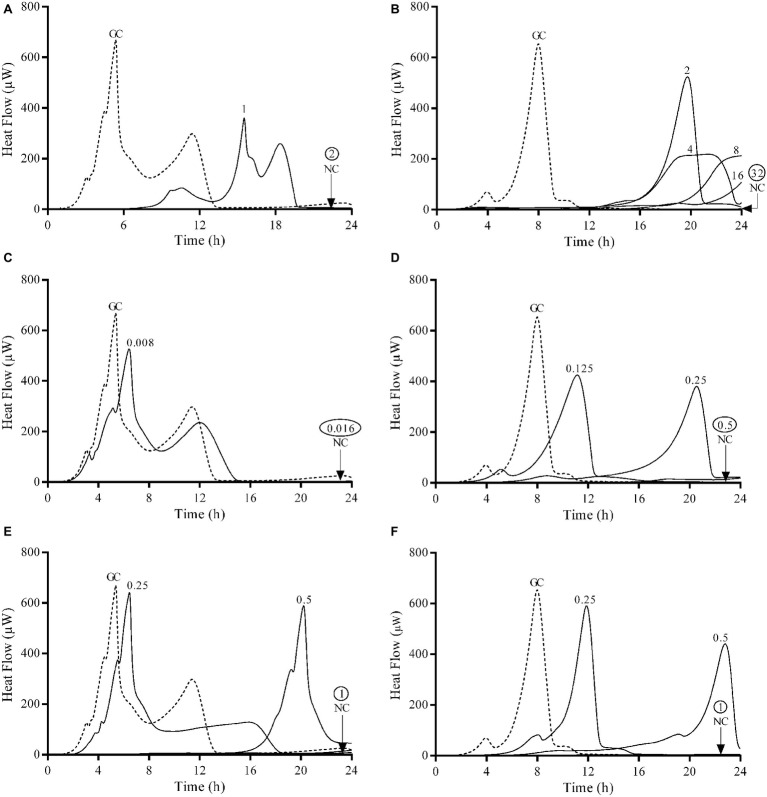
Microcalorimetry analysis of planktonic bacteria exposed to serial antibiotic concentrations for 24 h. Numbers represent concentrations (in μg/ml) of fosfomycin **(A,B)**, ciprofloxacin **(C,D)**, and gentamicin **(E,F)** against *E. coli* ATCC 25922 (left column) and *P. aeruginosa* ATCC 27853 (right column). Circled values represent the MHIC. GC, growth control; NC, negative control. Data of a representative experiment are reported.

**Figure 2:**
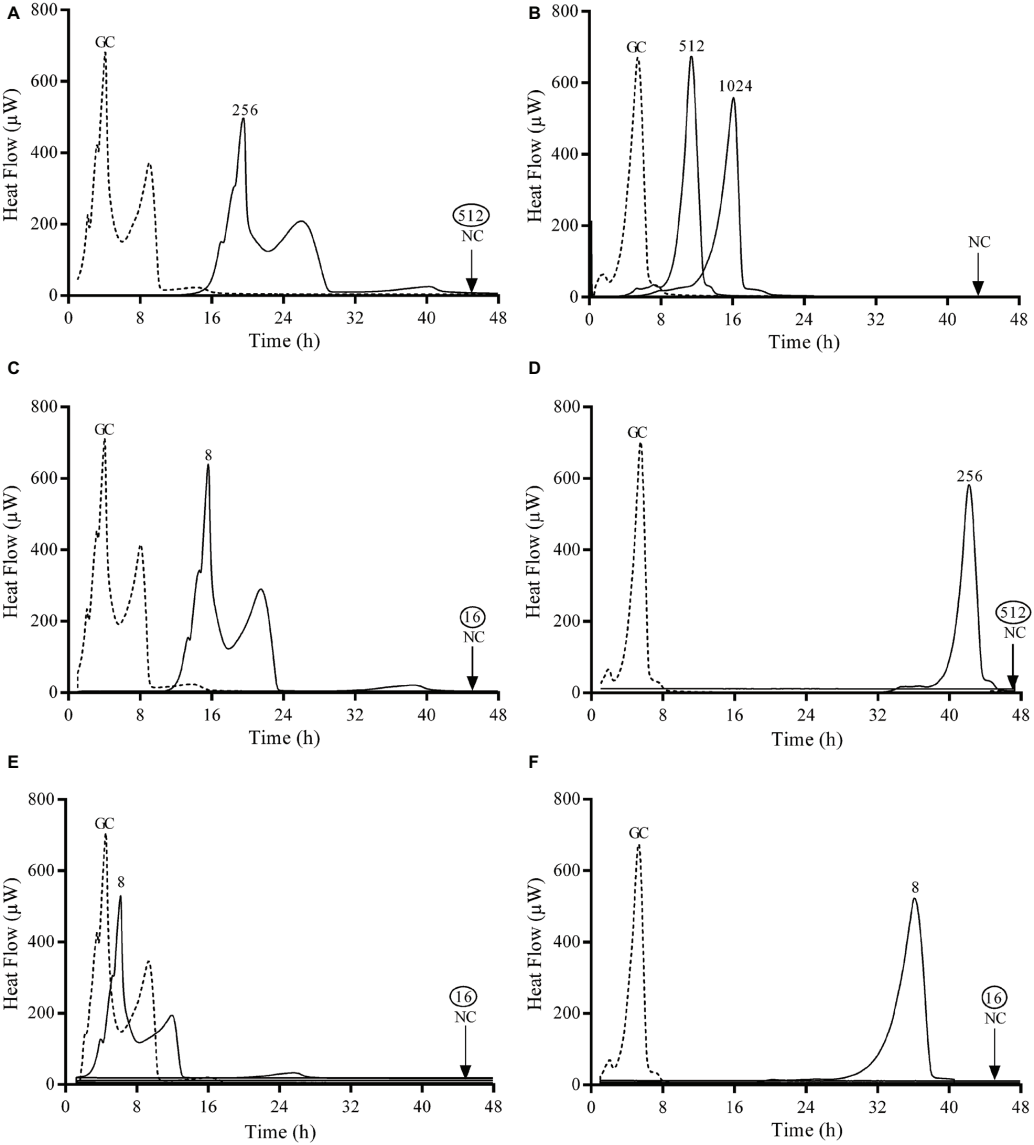
Microcalorimetry analysis of biofilm bacteria after exposure to serial antibiotic concentrations for 24 h. Numbers represent concentrations (in μg/ml) of fosfomycin **(A,B)**, ciprofloxacin **(C,D)**, and gentamicin **(E,F)** against *E. coli* ATCC 25922 (left column) and *P. aeruginosa* ATCC 27853 (right column). Circled values represent the MBBC. GC, growth control; NC, negative control. Data of a representative experiment are reported.

The antimicrobial activity of fosfomycin, ciprofloxacin, and gentamicin against planktonic and sessile *E. coli* and *P. aeruginosa* ATCC strains was assessed using BMC, Etest, IMC (Figures 1, 2) and by plating of sonication fluid. Table 1 summarizes the susceptibilities of both species.

**Table 1:** Antimicrobial susceptibility of planktonic and adherent *E. coli* and *P. aeruginosa* determined by conventional broth macrodilution (BMD), Etest, isothermal microcalorimetry (IMC), and sonication/colony-counting.

*E. coli* ATCC 25922						
**Antibiotic**	**Etest**	**BMD**		**IMC**		**Sonication**
	**MIC**	**MIC**	**MBC**	**MHIC**	**MBBC**	**MBEC**
Fosfomycin	1	2	4	2	512	512
Ciprofloxacin	0.012	0.008	0.016	0.016	16	16
Gentamicin	0.5	0.5	4	1	16	16
***P. aeruginosa* ATCC 27853**						
**Antibiotic**	**Etest**	**BMD**		**IMC**		**Sonication**
	**MIC**	**MIC**	**MBC**	**MHIC**	**MBBC**	**MBEC**
Fosfomycin	16	32	256	32	>1,024	>1,024
Ciprofloxacin	0.25	0.25	1	0.5	512	512
Gentamicin	1	0.5	4	1	16	16

*Concentration values are expressed in μg/ml*.

The observed MIC values evaluated by Etest and BMD were comparable to those obtained by IMC. Both ATCC strains were susceptible to all three tested antibiotics. Ciprofloxacin was the most active antibiotic against planktonic bacteria from both strains, followed by gentamicin. Fosfomycin showed a remarkable lower bactericidal activity against *P. aeruginosa* with MIC and MBC values 16 and 64 times higher respectively compared to *E. coli*.

Gentamicin was the most active antibiotic against the biofilm of both strains presenting a MBBC of 16 μg/ml (Figures 2E,F), whereas ciprofloxacin showed a notable higher anti-biofilm activity against *E. coli* (MBBC = 16 μg/ml) compared to *P. aeruginosa* (MBBC = 512 μg/ml) (Figures 2C,D). Fosfomycin exhibited a poor anti-biofilm activity against both tested ATCC strains (Figures 2A,B).

Results showed that the concentrations of antibiotics necessary to completely eradicate the biofilm (MBEC) of both ATCC strains correlated with the bactericidal concentrations observed by calorimetry (MBBC) for all the tested antibiotics.

### Anti-biofilm Activity of Combined Antibiotics Against *E. coli* and *P. aeruginosa* ATCC Strains

**Figure 3:**
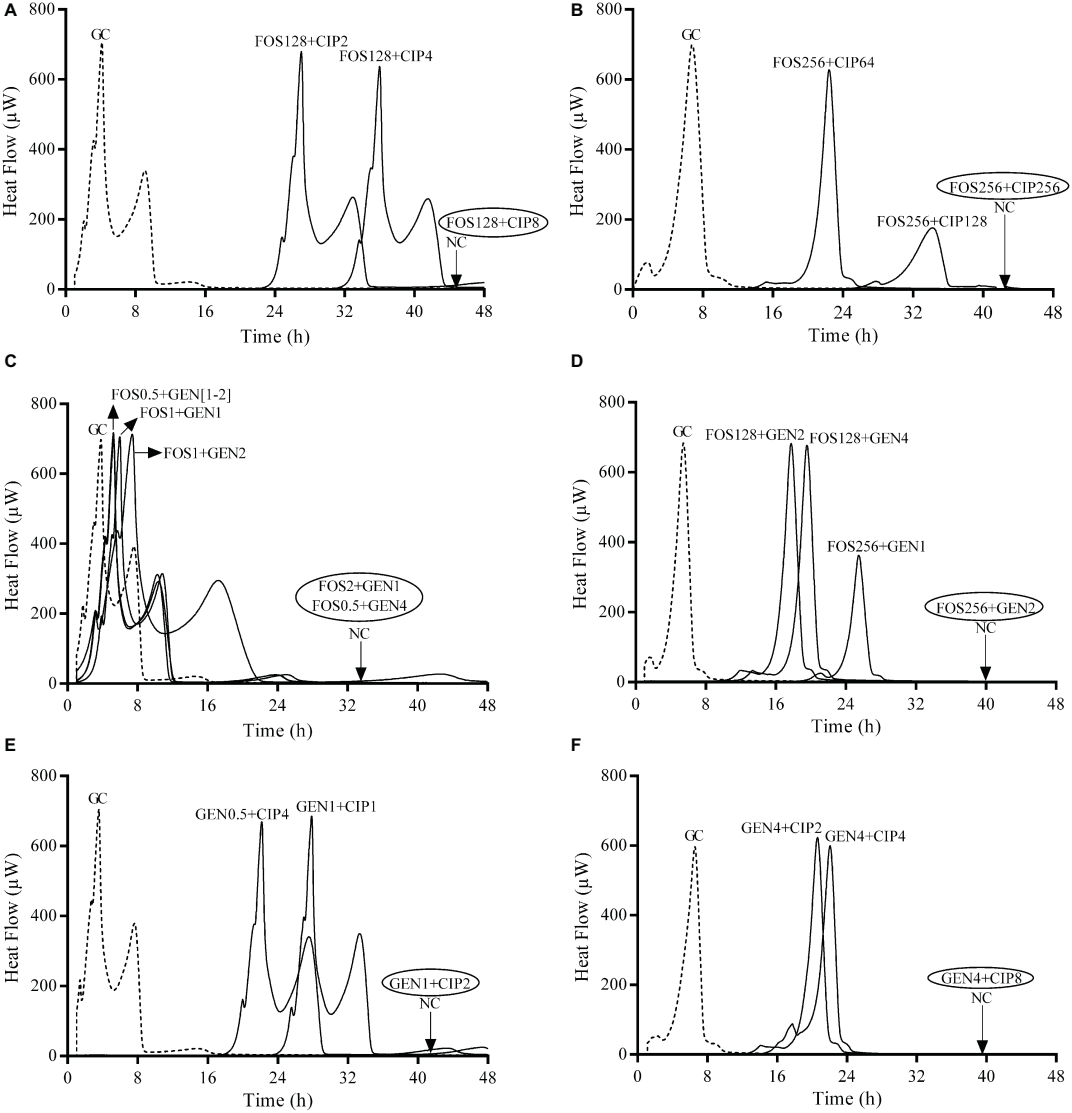
Evaluation of synergistic activity of paired antibiotics by IMC against *E. coli* ATCC 25922 **(A,C,E)** and *P. aeruginosa* ATCC 27853 **(B,D,F)** biofilms. Numbers represent concentrations (in μg/ml). Circled values represent the MBBC. GC, growth control; NC, negative control; FOS, fosfomycin; GEN, gentamicin; CIP, ciprofloxacin. Data of a representative experiment are reported.

The synergistic effect of two-pair antibiotics against biofilm of both ATCC strains was investigated by IMC combining fosfomycin/ciprofloxacin, fosfomycin/gentamicin, and gentamicin/ciprofloxacin. Results are summarized in Table 2. Calorimetric curves are depicted in Figure 3.

**Table 2:** MBBC and FBEC values for fosfomycin (FOS), ciprofloxacin (CIP), and gentamicin (GEN) in combination against *E. coli* and *P. aeruginosa*.

Antibiotic	*E. coli* (ATCC 25922)		*P. aeruginosa* (ATCC 27853)	
	MBBC (μg/ml)	FBEC (interpretation)	MBBC (μg/ml)	FBEC (interpretation)
FOS + CIP	128 + 8	0.75 (NS)	256 + 256	0.75* (NS)
FOS + GEN	2 + 1	0.06 (S)	256 + 2	0.38* (S)
GEN + CIP	1 + 2	0.19 (S)	4 + 8	0.26 (S)

MBBC, minimal biofilm bactericidal concentration; FBEC, fractional biofilm eradication concentration; S, synergism, NS, no synergism; *MBBC_FOS_ of *P. aeruginosa* was considered equal to 1,024 μg/ml for FBEC calculations.

The strongest synergistic effect was observed when gentamicin was combined with fosfomycin (FBEC = 0.06) against *E. coli* biofilm followed by the combination of gentamicin with ciprofloxacin, whereas these two antibiotic combinations showed similar synergistic effect against *P. aeruginosa* biofilm. Fosfomycin/ciprofloxacin combination did not show synergism against the biofilm of both strains.

### Antibiotic Susceptibility of *E. coli* and *P. aeruginosa* Clinical Strains

The MIC of *E. coli* and *P. aeruginosa* clinical strains to fosfomycin, ciprofloxacin, and gentamicin was determined by Etest. The results are summarized in Tables 3 and 4.

**Table 3:** MIC of *E. coli* by Etest.

Antimicrobial	Ec1	Ec2	Ec3	Ec4	Ec5	Ec6	Ec7	Ec8
Fosfomycin	0.19	0.094	0.125	0.064	0.75	0.25	128(R)	64(R)
Ciprofloxacin	0.016	0.016	0.008	0.008	0.008	8(R)	8(R)	0.008
Gentamicin	1	0.5	1	2	1	96(R)	1	96(R)

*MIC values are expressed in μg/ml; R, resistant (according to CLSI)*.

**Table 4:** MIC of *P. aeruginosa* by Etest.

Antimicrobial	Pa1	Pa2	Pa3	Pa4	Pa5	Pa6	Pa7
Fosfomycin	32	48	8	16	32	24	48
Ciprofloxacin	0.19	0.064	0.125	0.125	0.094	0.19	12(R)
Gentamicin	1.5	1.5	2	3	2	128(R)	1.5

*MIC values are expressed in μg/ml; R, resistant (according to CLSI)*.

*E. coli* and *P. aeruginosa* strains were considered susceptible to fosfomycin when MIC ≤64 μg/ml, to ciprofloxacin when MIC ≤1 μg/ml and to gentamicin when MIC ≤4 μg/ml according to CLSI (CLSI, 2015).

Most strains were susceptible to the tested antibiotics, except Ec6 (resistant to ciprofloxacin and gentamicin), Ec7 (resistant to fosfomycin and ciprofloxacin), Ec8 (resistant to fosfomycin and gentamicin), Pa 6 (resistant to gentamicin), and Pa 7 (resistant to ciprofloxacin).

Ciprofloxacin exhibited the lowest MIC in sensitive strains for both bacterial species (MIC range 0.008–0.25 μg/ml), whereas fosfomycin showed higher activity on susceptible strains of *E. coli* (MIC range 0.064–1 μg/ml) than *P.* aeruginosa (8–48 μg/ml). Gentamicin-susceptible strains from both species presented similar susceptible profile with a MIC range of 0.5–3 μg/ml.

### Synergistic Effect of Antibiotic Combinations Against *E. coli* and *P. aeruginosa* Clinical Strains

The same two-pair antibiotic combinations tested against the ATCC strains were used to evaluate their ability to eradicate biofilms from clinical strains by sonication/colony counting. Tables 5 and 6 summarize the results of the MBEC for single and combined antibiotics against *E. coli* and *P. aeruginosa* clinical strains.

**Table 5:** MBEC for fosfomycin (FOS), ciprofloxacin (CIP), gentamicin (GEN), and their combinations against *E. coli* clinical strains.

Strain	MBEC (FBEC, interpretation)					
	FOS	CIP	GEN	FOS + CIP	FOS + GEN	GEN + CIP
Ec1	16	4	16	4 + 2 (0.75, NS)	1 + 1 (0.125, S)	0.5 + 0.5 (0.16, S)
Ec2	4	64	8	0.5 + 2 (0.16, S)	0.5 + 1 (0.25, S)	1 + 2 (0.16, S)
Ec3	16	0.032	8	4 + 0.016 (0.75, NS)	2 + 1 (0.25, S)	2 + 0.016 (0.75, NS)
Ec4	8	0.032	8	2 + 0.016 (0.75, NS)	2 + 0.5 (0.31, S)	2 + 0.016 (0.75, NS)
Ec5	8	64	16	2 + 1 (0.27, S)	1 + 1 (0.19, S)	4 + 0.5 (0.26, S)
Ec6	16	>1,024	>1,024	>4 + 256* (>0.5, NS)	2 + 16* (0.14, S)	>256* + 256* (>0.5, NS)
Ec7	>1,024	>1,024	4	>256* + 256*(>0.5, NS)	>256* + 1 (>0.5, NS)	>1 + 256* (>0.5, NS)
Ec8	>1,024	8	>1,024	>256* + 2 (>0.5, NS)	>256* + 256*(>0.5, NS)	>256* + 2 (>0.5, NS)

MBEC, minimal biofilm eradication concentration (values are expressed in μg/ml). FBEC, fractional biofilm eradication concentration; S, synergism; NS, no synergism; *MBBC was considered equal to 1,024 μg/ml for FBEC calculations.

**Table 6:** MBEC for fosfomycin (FOS), ciprofloxacin (CIP), gentamicin (GEN), and their combinations against *P. aeruginosa* clinical strains.

Strain	MBEC (FBEC, interpretation)					
	FOS	CIP	GEN	FOS + CIP	FOS + GEN	GEN + CIP
Pa1	>1,024	4	8	128* + 1 (0.38, S)	128* + 2 (0.375, S)	2 + 1 (0.5, S)
Pa2	>1,024	32	32	256* + 16 (0.75, NS)	64* + 4 (0.19, S)	4 + 2 (0.19, S)
Pa3	>1,024	16	16	128* + 2 (0.25, S)	128* + 1 (0.19, S)	1 + 1 (0.13, S)
Pa4	>1,024	8	16	32* + 2 (0.28, S)	64* + 1 (0.13, S)	4 + 1 (0.38, S)
Pa5	>1,024	256	128	256* + 128 (0.75, NS)	256* + 64 (0.75, NS)	16 + 32 (0.25, S)
Pa6	>1,024	16	>1,024	64* + 4 (0.31, S)	>256* + 256*(>0.5, NS)	>256* + 4 (>0.5, NS)
Pa7	>1,024	>1,024	16	>256* + 256*(>0.5, NS)	>256* + 4 (>0.5, NS)	>4 + 256*(>0.5, NS)

MBEC, minimal biofilm eradication concentration (values are expressed in μg/ml). FBEC, fractional biofilm eradication concentration; S, synergism; NS, no synergism; *MBBC was considered equal to 1,024 μg/ml for FBEC calculations.

The biofilm susceptibility to each antibiotic varied widely among clinical isolates. Among the eight tested *E. coli* isolates, synergism based on fosfomycin/ciprofloxacin combinations was observed in two isolates (25%), while the combinations fosfomycin/gentamicin and gentamicin/ciprofloxacin resulted synergistic in six (75%) and three isolates (37.5%), respectively.

Moreover, the fosfomycin/gentamicin combination showed a synergistic effect against Ec6, a gentamicin-resistant but fosfomycin-susceptible *E. coli* strain, whereas the synergistic effect was not observed when the fosfomycin/gentamicin combination was tested against Ec7, a fosfomycin-resistant but gentamicin-susceptible *E. coli* strain.

On the other hand, the synergism of gentamicin/ciprofloxacin was observed in five *P. aeruginosa* isolates (71.4%), while four isolates (57.1%) were susceptible to the combination of either fosfomycin/ciprofloxacin or fosfomycin/gentamicin.

## Discussion

Fluoroquinolones are the first choice as anti-biofilm antibiotics for the treatment of GN-PJI (Boyle et al., 2019). However, emergence and spread of resistance to fluoroquinolones and aminoglycosides has decreased the existing treatment options for GN infections (Tucaliuc et al., 2015). Combination therapy with fosfomycin has been recommended, particularly against fluoroquinolone resistant organisms (Boyle et al., 2019). Nonetheless, there is a lack of systematic studies investigating antibiotic combinations under the same experimental settings on GN biofilms. In this study, we have generated original data showing synergistic activity of two-pair antibiotic combinations against either *E. coli* or *P. aeruginosa* biofilms *in vitro*.

Conventional (Etest, BMD and colony counting) and nonconventional (IMC) laboratory tests were applied to evaluate the susceptibility to antibiotics of either planktonic or biofilm bacteria. As seen also in previous studies (Gonzalez Moreno et al., 2017, 2019), the MHIC values obtained by IMC showed consistency to the MIC values obtained by BMD and Etest, proving the reliability of IMC for antimicrobial testing on large scale. Moreover, the concentrations of antibiotics needed to eradicate biofilms were analogous to those showing biofilm bactericidal activity by IMC.

As already reported for many microorganisms (Stewart, 2015), in our study, the eradication of GN biofilms required considerably higher concentrations of all three tested antibiotics (4 to 2,723-fold higher) compared with the killing of their planktonic bacteria. Fosfomycin had no anti-biofilm activity against *P. aeruginosa* strains despite the use of high concentrations of antibiotic (up to 1,024 μg/ml) (Table 6). These results suggest that outcomes obtained on planktonic cells cannot be transferred to biofilms, underling the importance of developing standardize methods to evaluate antimicrobial activity on biofilms (Macia et al., 2014). In our work, we employed IMC in combination with sonication as reliable methods for the *in vitro* analysis of bacterial biofilms (Butini et al., 2018).

In biofilm infections, where antimicrobial monotherapies are not effective or not applicable due to the rapid development of antibiotic resistance (Wu et al., 2015; Greimel et al., 2017; Xu et al., 2018), the use of antibiotic combinatorial therapies has been shown to be particularly relevant due to the potential synergy between drugs (Wu et al., 2015). Therefore, we also investigated the *in vitro* synergistic activity of paired antibiotics against *E. coli* and *P. aeruginosa* ATCC strains and clinical isolates.

A study limitation is the lack of a full chequerboard analysis for antibiotic combinations, which could bring more insights on the synergistic/antagonistic activity. We evaluated only concentrations of antibiotics that could reveal a synergistic effect based on the MBEC values of the single antibiotics to be combined. For MBEC >1,024 μg/ml, a fixed value of 1,024 μg/ml was considered for the calculation of the FBEC index, thus some combinations which were interpreted as not synergistic could turn out to have a synergistic effect considering higher MBEC values. However, with this approach, the observed positive synergistic effects of antibiotic combinations are certain and usually presenting considerably lower MBEC values compared to the MBEC values of single antibiotics, which are difficult to reach in clinical practice.

The three tested antibiotic combinations showed synergistic activity, to varying degrees, against different clinical strains from both species partially differing from the results observed with the ATCC strains. The considerable reduction of MBEC values with antibiotic combinations ranging from 2-fold to 16-fold in case of *P. aeruginosa* strains or 2-fold to 128-fold in case of *E. coli* strains compared to single drug was predominantly in the range, which is achievable by intravenous or oral antibiotic administration (Dijkmans et al., 2017; Thabit et al., 2019).

Even though several studies have reported that fosfomycin showed an estimable synergistic effect, among others, with gentamicin and ciprofloxacin against *P. aeruginosa* planktonic cells (Kastoris et al., 2010), there is limited evidence for these combinations against biofilms. The fosfomycin/ciprofloxacin combination has being shown to be effective against *P. aeruginosa* biofilms in other experimental set-ups (Xiong et al., 1995; Mikuniya et al., 2005), whereas we could not find studies investigating the fosfomycin/gentamicin combination. Nonetheless, previous studies have shown synergistic effect when fosfomycin was combined with other aminoglycosides (Cai et al., 2009; Anderson et al., 2013) as well as other fluoroquinolones (Kumon et al., 1995; Monden et al., 2002; Mikuniya et al., 2005, 2007) against *P. aeruginosa* biofilms.

Some authors propose that fosfomycin alters the membrane permeability of *P. aeruginosa* by affecting cell wall synthesis, which should lead to enhanced uptake of the fluoroquinolone ofloxacin (Monden et al., 2002). On the other hand, one study has suggested that the role of ciprofloxacin is thought to be related to damage of the outer membrane, enhancing fosfomycin penetration (Yamada et al., 2007). Regarding permeability in biofilms, it was reported that ciprofloxacin had a higher penetration rate (>75%) than gentamicin (73%) in *P. aeruginosa* biofilms (Abdi-Ali et al., 2006), but showed a similar kinetic of penetration than fosfomycin into the bacterial biofilm of both, *E. coli* and *P. aeruginosa* species (Rodríguez-Martínez et al., 2007). Based on these studies, it can be argued that all three antibiotics are able to penetrate well into biofilms. Thus, it could be hypothesized that the differences observed on the ability of each antibiotic combination to exert a synergistic effect might be attributed to other factors, such as killing of persister cells as proposed for streptococci in combinations including gentamicin (Gonzalez Moreno et al., 2017), rather than the enhancement of penetration. Further studies are required to clarify the underlying mechanism of their synergistic effect on biofilms.

The fosfomycin/gentamicin combination was the most active against *E. coli* strains. These results also correlate with the findings from Corvec et al., where fosfomycin plus gentamicin presented a significant high cure rate in an *in vivo* foreign-body infection model (Corvec et al., 2013). Moreover, the fosfomycin/gentamicin combination showed a synergistic effect toward a gentamicin-resistant strain, but the same combination was not synergistic toward a fosfomycin-resistant strain. Conventionally, the mechanism of action of gentamicin has been considered at the 30S ribosomal level. Nevertheless, some authors have suggested that gentamicin has two potentially lethal effects on Gram-negative cells, one being the inhibition of protein synthesis and the other one being the surface perturbation (Kadurugamuwa et al., 1993a,b). Thus, a bacteriolysis effect mediated through perturbation of the cell surface by gentamicin could explain the synergism observed by the fosfomycin/gentamicin combination toward a gentamicin-resistant strain. It could be speculated that, in the case of a gentamicin-resistant but fosfomycin-susceptible strain, while fosfomycin can act against susceptible bacterial cells, gentamicin could also actively target resistant bacterial cells through bacteriolysis, resulting in complete biofilm eradication. However, in the case of a gentamicin-susceptible but fosfomycin-resistant strain, no synergistic effect is observed because only gentamicin can act against bacteria, whereas fosfomycin becomes ineffective. The two antimicrobial effects of gentamicin might also explain why combinations with this antibiotic seem to be particularly effective. Still, elucidations for synergistic effect based on planktonic findings would need to be confirmed also for biofilms.

In conclusion, the use of fosfomycin in combination with gentamicin seems to be a promising therapeutic approach against *E. coli* biofilm related infections. Nevertheless, against both Gram-negative species, combination of gentamicin with ciprofloxacin represents the most optimal treatment option. Further *in vivo* and clinical studies are essential to define the potential treatment regimen based on the combination of these two antibiotics. Moreover, our study presents IMC as a sensitive technique to provide reliable data on as important field of clinical microbiology as it is the screening for biofilm-eradicating approaches.

## Data Availability Statement

The datasets generated for this study are available on request to the corresponding author.

## Ethics Statement

The clinical isolates were used from the biobank collection, which is part of the prospective institutional PJI cohort. The study was approved by the Institutional Ethical Committee (EA1/040/14) and was conducted in accordance with the most recent iteration of the Declaration of Helsinki. According to the ethical approval, participants’ informed consent was waived and all data were pseudonymized.

## Author Contributions

LW, MG, MD, TT, and AT conceived and designed the experiments. LW performed the experiments. LW and MG analyzed the data. MG and LW drafted the manuscript, with the contribution of MD and AT. All authors reviewed and revised the final drafts of this manuscript.

### Conflict of Interest

The authors declare that the research was conducted in the absence of any commercial or financial relationships that could be construed as a potential conflict of interest.
